# Stress, Cortisol and NR3C1 in At-Risk Individuals for Psychosis: A Mendelian Randomization Study

**DOI:** 10.3389/fpsyt.2020.00680

**Published:** 2020-07-10

**Authors:** Anton Iftimovici, Oussama Kebir, Qin He, Thérèse M. Jay, ICAAR Study Group, Isabelle Amado, Guy A. Rouleau, Marie-Odile Krebs, Boris Chaumette

**Affiliations:** ^1^ Institut de Psychiatrie et Neurosciences de Paris, INSERM UMR 1266, Laboratoire de Physiopathologie des Maladies Psychiatriques, Université de Paris, GDR3557-Institut de Psychiatrie, Paris, France; ^2^ NeuroSpin, Atomic Energy Commission, Gif-sur-Yvette, France; ^3^ GHU Paris Psychiatrie et Neurosciences, Paris, France; ^4^ Department of Neurology and Neurosurgery, Montreal Neurological Institute, McGill University, Montreal, QC, Canada; ^5^ Department of Psychiatry, McGill University, Montreal, QC, Canada

**Keywords:** ultra-high risk of psychosis, stress, cortisol, hypothalamic–pituitary–adrenal axis, Mendelian randomization, expression quantitative trait locus, genome-wide analysis study

## Abstract

**Introduction:**

The emergence of psychosis in at-risk individuals results from interactions between genetic vulnerability and environmental factors, possibly involving dysregulation of the hypothalamic-pituitary-adrenal axis. Hypercorticism was indeed described in schizophrenia and ultra-high-risk states, but its association with clinical outcome has yet to be demonstrated. The impact of stress through cortisol may vary depending on the expression level of genes related to the stress pathway.

**Methods:**

To test this hypothesis, we selected *NR3C1*, the gene encoding the glucocorticoid receptor, and modeled through logistic regression how its peripheral expression could explain some of the risk of psychosis, independently of peripheral cortisol levels, in a French longitudinal prospective cohort of 133 at-risk individuals, adjusted for sex, age, cannabis, and antipsychotic medication intake. We then performed a genome-wide association analysis, stratified by sex (55 females and 78 males), to identify *NR3C1* expression quantitative trait loci to be used as instrumental variables in a Mendelian randomization framework.

**Results:**

*NR3C1* expression was significantly associated with a higher risk of conversion to psychosis (OR = 2.03, p = 0.03), independently of any other factor. Cortisol was not associated with outcome nor correlated with *NR3C1*. In the female subgroup, rs6849528 was associated both with *NR3C1* mRNA levels (p = 0.015, Effect-Size = 2.7) and conversion (OR = 8.24, p = 0.03).

**Conclusions:**

For the same level of cortisol, *NR3C1* expression increases psychotic risk, independently of sex, age, cannabis, and antipsychotic intake. In females, Mendelian randomization confirmed *NR3C1*’s effect on outcome to be unbiased by any environmental confounder.

## Introduction

The concept of schizophrenia has moved from a chronic to a progressive illness that typically emerges during late adolescence and goes through several stages: early vulnerability, at-risk mental state, first episode of psychosis (FEP), and finally, chronic disease (1, 2). The at-risk state includes subjects with psychotic symptoms that are either attenuated or not frequent enough to allow a diagnosis of FEP, or who have a genetic risk and present with nonspecific functional decline. Only up to a third of at-risk subjects might convert to FEP after 3 years and the reasons for this differential outcome are yet to be understood (3). According to the main hypothesis in the field, the emergence of psychotic symptoms could imply an interaction between genes and environment, and could be mediated through epigenetic (4) and transcriptomic processes (5). The biological response to stress has been hypothesized to play a role in pathophysiology. Abnormal cortisol levels have indeed been suggested at each stage: increased basal cortisol in subjects at-risk (6) or with schizophrenia (7), a possibly blunted cortisol awakening response in FEP and in schizophrenia (8), and an attenuated cortisol response to acute psychosocial stress in at-risk subjects (9). Cortisol levels also have been proposed to predict the prognosis in the at-risk individuals (10). However, relatively small sample sizes and heterogeneous measures and outcomes mean that these findings need to be interpreted with caution. Moreover, our recent meta-analysis did not confirm the association between morning salivary cortisol levels and conversion to psychosis (6). The frequency of stress-related dysregulation in the at-risk group as a whole might indeed not allow cortisol by itself to discriminate between future converters and non-converters.

Further investigations of the stress pathway, at a molecular and genetic levels, are therefore warranted. To this purpose, confounders such as sex or environmental factors need to be controlled, as they might account for cortisol’s observed weak predictive power. Cortisol awakening response could be significantly lower in males with FEP compared to male controls and affected females (11). Sex differences have also been suggested in morning cortisol levels in the at-risk stage (12). This moderating effect of sex might further be seen at the molecular level, on the glucocorticoid nuclear receptor *NR3C1*, which acts as a transcription factor that binds to glucocorticoid response elements and regulates gene expression upon stress. Sex-specific effects of negative environment on *NR3C1* regulatory regions’ methylation have been observed (13), as well as sex-specific upregulation of *NR3C1* transcription under acute stress in animal models (14). In humans, it has recently been suggested that epigenetic mechanisms, including *NR3C1* regulation, might differ between males and females (15), with a sex-dependent role of *NR3C1*’s methylation in depression, but also a sex-specific effect of allelic variation of the *NR3C1* gene (16, 17).

Moreover, *NR3C1* expression could be of interest in psychosis, not only through its relationship with cortisol, but also as a possible direct marker. One study, although limited in sample size, suggested that *NR3C1* mRNA might be decreased in the dorso-lateral prefrontal cortex of schizophrenia cases, relative to controls, while among schizophrenia cases, it might be increased in suicide-positive vs suicide-negative subjects (18). Furthermore, anxiety and depression are important prodromal symptoms in the emergence of psychosis (2), and allelic variations in the *NR3C1* gene have been found associated with depression, with or without psychotic features (19, 20), as well as with cognitive deterioration, independently of cortisol levels (20). However, there is not, to our knowledge, any *NR3C1* gene expression (mRNA or protein) study in the context of emergence of psychosis.

**Figure 1:**
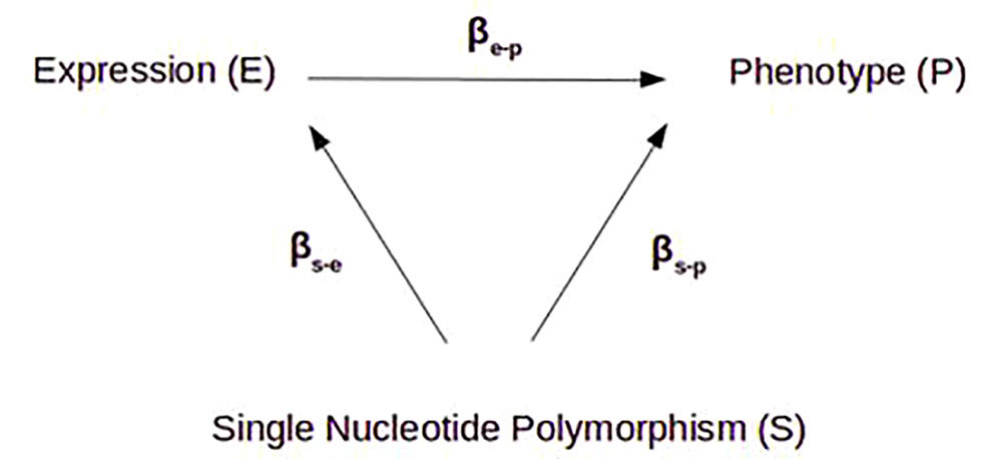
Principle of Mendelian randomization. In a purely causal model, the variation of gene expression (e) is fully determined by an eQTL SNP (s), which itself has no effect (β) on phenotype (p) except through that gene’s expression, so βs-e x βe-p = βs-p. By identifying the different β, we can estimate the effect of gene expression on the phenotype (explained variance), free of other potential confounding factors.

In this context, we hypothesized that, in at-risk individuals, *NR3C1* peripheral expression may explain some of the risk of psychosis, independently of cortisol levels, and adjusted by sex, age, cannabis and antipsychotic medication intake. To shore up this argument, we considered a Mendelian randomization analysis in order to account for all possible unknown environmental factors. In this analysis, the effect of an “exposure” variable (here, the level of gene expression) on an “outcome” variable (here, conversion to psychosis) is tested through the use of an “instrument” that explains exposure independently of confounding factors (e.g. environmental biases): stratifying a population using the instrumental variable thus allows to test the unbiased effect of the exposure on the outcome (21). A genetic variant representing an expression quantitative trait locus (eQTL) of this gene could serve as such an instrumental variable, because it does not depend on the environment (**Figure 1**) (22). In our data, if an eQTL explained the variation of *NR3C1* expression, and if this expression level was associated with conversion to psychosis, then stratifying at-risk individuals on this eQTL could highlight the differences in conversion rates that are only due to *NR3C1* expression levels, independently of any non-genetic confounders.

**Figure 2:**
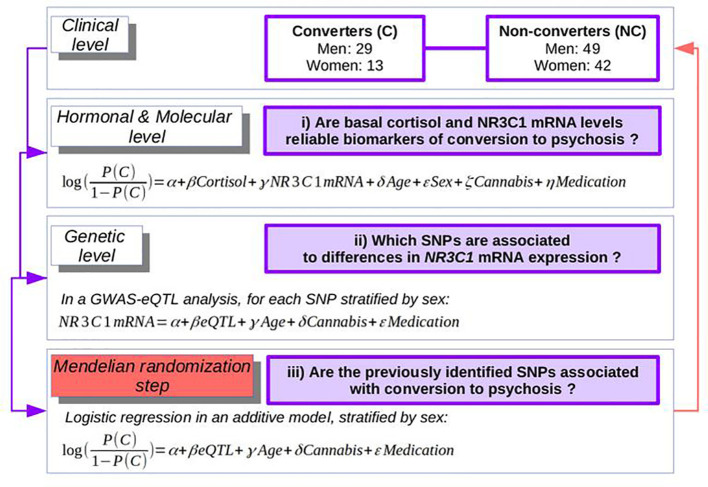
The strategy of the analyses: from clinical to hormonal, molecular, and genetics levels.

Using a French longitudinal prospective cohort of individuals at-risk for psychosis, presenting attenuated or prodromal symptoms [ICAAR, previously described (23)], with data on cortisol levels, *NR3C1* expression levels, and whole-genome genotyping, we thus tested the following hypotheses in a Mendelian randomization framework (**Figure 2**). i) At the hormonal and molecular level, are basal cortisol and *NR3C1* expression reliable biomarkers of conversion to psychosis? ii) At the genetic level, does a genome-wide analysis study (GWAS), stratified by sex to account for genotype-by-sex interactions (16, 17), identify any eQTL that can explain *NR3C1* mRNA levels? iii) Finally, would such an eQTL be associated with psychosis, thus confirming the environmentally unbiased effect of *NR3C1* on risk of psychosis? Last, as secondary outcomes, we tested if *NR3C1* expression is associated with functional or depressive outcomes or more specifically with psychotic features.

## Material and Methods

### Population

Participants were recruited through the French ICAAR cohort (PHRC AOM-07-118, promoted by Hôpital Sainte-Anne) among help-seeking individuals (16 to 30 years old) consecutively referred to the Adolescent and Young Adult Assessment Centre (Service Hospitalo-Universitaire, Hôpital Sainte-Anne, Paris, France) between 2009 and 2014 (23). All help-seeking individuals were examined at baseline and after 1 year follow-up with the Comprehensive Assessment of at-risk mental state, CAARMS (24), in its translated version (25), by specifically trained psychiatrists. After baseline assessment, a consensus meeting for best estimated diagnosis was held, and help-seekers were classified as at-risk for psychosis stage IA or stage IB according to the staging model distinction (26). Were included in the analysis all at-risk stage IA and stage IB individuals, for which clinical, biological and genetic data was available. Stage IA included patients with mild or non-specific symptoms of psychosis or severe mood disorder, and mild functional change. Stage IB included patients with moderate subthreshold symptoms and moderate functional change. Inclusion criteria were alterations in global functioning (Social and Occupational Functioning Assessment Scale score < 70) during the past year, which were associated with psychiatric symptoms and/or subjective cognitive complaints. Exclusion criteria included manifest symptoms of psychosis (fulfilling DSM-IV criteria), or other established psychiatric diagnoses (pervasive developmental disorder, bipolar disorder, obsessive compulsive disorder), serious or non-stabilized somatic and neurological disorders, head injury and IQ below 70. Psychotic conversion was characterized using the CAARMS-defined psychosis onset threshold (i.e., supra-threshold psychotic symptoms—thought content, perceptual abnormalities and/or disorganized speech—present for more than 1 week). Individuals who reached the threshold during the follow-up were considered as converters and individuals who recovered or displayed persistent subthreshold symptoms were called non-converters. Additionally, each individual underwent clinical assessments including the Social and Occupational Functioning Assessment Scale (SOFAS), the Positive And Negative Syndrome Scale (PANSS) and the Montgomery-Åsberg depression rating scale (MADRS), cannabis intake in the last month, and antipsychotic treatment summarized by the chlorpromazine equivalent doses (references for the computation of chlorpromazine equivalent doses are available in **Supplementary Table 1**). The population demographic and clinical characteristics at baseline are described in **Table 1**. We used the conversion status available after 1-year follow-up in order to have an outcome that was closest to biological sampling.

**Table 1:** Demographic, clinical characteristics, and cortisol measures of the male and female datasets at baseline.

Measure	Males			Females		
	Converters mean ± std	Non-Converters mean ± std	P-value	Converters mean ± std	Non-Converters mean ± std	P-value
Number of subjects	29	49		13	42	
Age at baseline	19.9 ± 2.5	21.4 ± 3.4	0.112	22.1 ± 3.7	21.1 ± 4.1	0.340
***Symptoms***						
PANSS total	75.9 ± 15.6	67.3 ± 20.2	**0.041**	66.7 ± 22.0	61.7 ± 13.7	0.307
MADRS	21.8 ± 8.4	18.2 ± 9.9	**0.038**	21.4 ± 8.9	22.9 ± 9.8	0.368
SOFAS	43.9 ± 8.8	48.1 ± 11.6	1.0	51.9 ± 9.4	48.7 ± 8.4	0.156
***Treatment***						
Antipsychotic use: % of patients	34.5 (10/29)	22.5 (11/49)	0.371	7.7 (1/13)	19 (8/42)	0.590
Chlorpromazine equivalent (mg)*	75.1 ± 37.6	138 ± 73.1	0.071	462 ± 0.0	77.4 ± 44.4	0.217
***Substance use (last month)***						
Tobacco use: % of patients	48.3 (14/29)	30.6 15/49	**0.046**	54 (7/13)	38 (16/42)	0.095
Cannabis use: % of patients	34.5 (10/29)	16.3 (8/49)	**0.01**	61.5 (8/13)	12 (5/42)	7.7 *10^–9^
***Cortisol measures***						
at 7:00 am (C1)	9.0 ± 6.3	8.4 ± 4.8	0.984	8.1 ± 4.2	9.7 ± 5.7	1.0
at 9:00 am (C2)	7.4 ± 3.9	9.5 ± 4.4	0.894	8.4 ± 1.5	10.4 ± 6.8	1.0
at 12:00 pm (C3)	5.3 ± 3.0	5.2 ± 3.6	0.941	5.3 ± 5.7	4.6 ± 2.5	1.0
at 5:00 pm (C4)	3.6 ± 2.9	4.3 ± 2.6	0.989	2.1 ± 1.2	2.3 ± 1.1	1.0

PANSS, Positive And Negative Syndrome Scale.

MADRS, Montgomery-Åsberg depression rating scale.

SOFAS, Social and Occupational Functioning Assessment Scale.

C1 to C4 are the four times of cortisol measures: 7am, 9am, 12pm, 5pm, respectively.

*References for the computation of each chlorpromazine equivalent are available in **Supplementary Table 1**. P-values < 0.05 are in bold.

### Cortisol Measures

Salivary cortisol was collected using a synthetic swab at four time-points during the day after initial enrollment. Cortisol was measured at 7:00 am (C1), 9:00 am (C2), 12:00 pm (C3), and 5:00 pm (C4). A range of ± 1 h was accepted for each time of sampling. None of the subjects worked night shifts. The saliva samples were stored at -20°C until analysis. After thawing, saliva samples were centrifuged at 2000 g for 10 min, which resulted in a clear supernatant of low viscosity; 100 μl of saliva were used for duplicate analysis of each sample. Cortisol measurement was done using a competitive solid phase time-resolved fluorescence immunoassay with fluorometric end point detection (DELFIA) conducted by Cortisollabor, University of Trier, Department of Clinical and Physiological Psychology, Trier, Germany (27). The inter-assay coefficient of variation was 8.6% and intra-assay coefficient of variation was 4.3% as previously reported (6).

### Gene Expression

Total RNA was extracted and purified from blood samples (PAXgene tubes) using a QIAcube robot and PAXgene Blood RNA kit (QIAGEN) according to the manufacturer’s protocol. Quality control was done using LabChip GX (Perkin Elmer, Waltham USA). The full quantitative PCR (qPCR) protocol has been described in Chaumette et al. (5). Briefly, complementary DNA (cDNA) synthesis was performed using Reverse Transcription Master Mix from Fluidigm^®^ according to the manufacturer’s protocol with random primers using a Nexus thermocycler (Eppendorf). Specific target pre-amplification was performed using a Fluidigm^®^ PreAmp Master Mix at 12 cycles. Real time PCR was performed on the qPCR-HD-Genomic Paris Centre platform, using BioMark™ HD System, GE Dynamic Arrays (Fluidigm) and TaqMan^®^ Gene Expression assays (Life Technologies, ThermoFisher). Thermal conditions for qPCR were: 25°C for 30 min and 70°C for 60 min for thermal mix; 50°C for 2 min and 95°C for 10 min for hot start; 40 cycles at 95°C for 15 s and 60°C for 1 min. Data were processed by automatic threshold, with linear derivative baseline correction using BioMark Real-Time PCR Analysis Software 4.0.1 (Fluidigm). The quality threshold was set at the default setting of 0.65. Normalization was done using the GAPDH rate followed by a livak normalization with a transformation by the 2ΔΔCT method (28) providing the relative mRNA expression level of *NR3C1* in each sample. Moreover, we checked on the Genevestigator platform (https://genevestigator.com), using the RefGenes tool (29), that *GAPDH* was among the top 10 genes with an expression that was both stable, and in *NR3C1* ranges, for it to be a good reference for normalization (**Supplementary Figure 1**).

### Genotyping Data

In the ICAAR cohort, 102 Caucasian individuals have been genotyped using the Infinium PsychArray-24 v1.2 BeadChip (Illumina). This chip was designed by the Psychiatric Genomic Consortium and is enriched for polymorphisms relevant for psychiatric diseases. Single Nucleotide Polymorphisms (SNPs) annotation was given by the Illumina annotation file. Plink v2.0 (www.cog-genomics.org/plink/2.0/) was used for quality control and association analyses. The quality control excludes samples with less than 90% genotyping rate (mind > 0.1), SNPs with a minor allele frequency less than 1% (maf < 0.01) and SNPs that were not genotyped in at least 60% of the sample (geno > 0.4). No sample was excluded during the quality control; after filtering, 306,841 SNPs remained to be analyzed in male samples and 300,732 in female samples. Linkage disequilibrium and haplotype blocks were analyzed with Haploview.

### Statistical Analysis

Statistical analyses of basal cortisol level and gene expression in ICAAR cohort were performed using Python 3.7.2 and R 3.6.2. Group distributions of quantitative values were compared with a non-parametric Mann-Whitney Wilcoxon rank sum test. Comparisons of multiple ordinal categorical groups were made with a Chi-squared test for proportions. Correlations were calculated using Spearman’s test. For demographic and clinical comparisons between groups, the Bonferroni corrected threshold was at 0.004. However, to be more stringent in detecting the potential confounders at baseline, we considered the uncorrected p-value threshold of 0.05 in order not to ignore any clinical difference that could bias the association between *NR3C1* expression and conversion, and for which Mendelian randomization would be needed. All measures were standardized using the mean and standard deviation in all the cohort. The reported effect-sizes (ES) were calculated with Hedge’s g. In our cohort of 133 subjects, we first regressed the odds of conversion, OR, as follows (**Figure 2**):

(I) OR=α+βCortisol+γNR3C1mRNA+δAge+εSex+ζCannabis+ηMedication

A second model using a linear regression on the same combination of explanatory variables was applied to test their association with depression (MADRS scale) and functional outcomes (SOFAS scale). Then for each SNP, stratified by sex, we applied a linear regression as follows:

(II) NR3C1mRNA=α+βeQTL+γAge+δCannabis+εMedication.

Finally, we applied a logistic regression to model the odds of conversion in the female group as follows:

(III) OR=α+βeQTL+γAge+δCannabis+εMedication.

#### Random Permutation Analysis and Bootstrapping

In order to derive robust non-parametric p-values for each Wilcoxon test, we randomly permuted the assignment of values to the groups, and repeated the statistical test 10,000 times. We then computed how many times a p-value was smaller or equal to the observed one. The reported p-value was calculated as the ratio of this number to the total number of tests done (10,000). The 95% confidence intervals (95% CI) were computed by bootstrapping, where the variance of means from each group was estimated by random sampling with replacement. This prevents any inference on the statistical distribution of the population.

#### Mendelian Randomization Analysis

The Mendelian randomization analysis follows the steps of a two-stage least squares (2SLS) regression analysis. First, the effect of gene expression (e) on phenotype (p) was estimated by >logistically regressing the log-odds of conversion on cortisol levels, *NR3C1* mRNA levels, age, sex, cannabis and antipsychotic intake. This gives a βe-p parameter for the effect of *NR3C1* mRNA on conversion. Second, using Plink v2.0 (30), we performed a GWAS eQTL analysis stratified by sex. *NR3C1* mRNA levels were used as the quantitative trait, linearly regressed on the alleles of each SNP in the GWAS, giving a βs-e parameter that estimates the effect of SNP eQTLs on expression (e). The usual false discovery rate (FDR) threshold of 0.05 was lowered to 0.025 to account for the sex stratification. Last, we measured the effect of the SNP we found (s) on phenotype (p) by applying logistic regression to explain the log-odds of conversion by the alleles of the SNP, giving a βs-p parameter (**Figure 1**). Its significance would confirm the effect of mRNA levels on the risk of conversion, independently from non-genetic confounders (**Figure 2**). An additive model was applied for the SNPs’ alleles.

## Results

### NR3C1 Expression but not Cortisol Levels are Associated with Conversion to Psychosis

At baseline, converters and non-converters were comparable in age, antipsychotic treatment and clinical scales, except for male converters who exhibited higher total PANSS and MADRS scores than male non-converters (p = 0.041 and 0.038, respectively). In both male and female groups, converters showed a higher cannabis use than non-converters (p = 0.010 and p = 7.7*10⁻⁹ respectively). Male converters smoked more tobacco than male non-converters (p = 0.046). None of the cortisol levels at any 4 times of the day were significantly different between converters and non-converters in either group (**Table 1**). None of the cortisol levels were significantly associated with *NR3C1* expression (**Supplementary Figure 2**).

**Figure 3:**
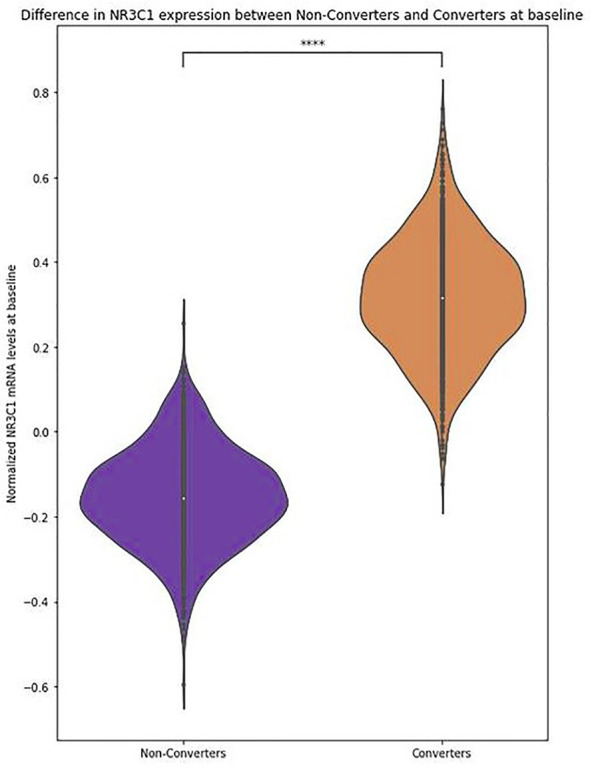
NR3C1 expression levels are higher in converters than in non-converters.

In model (I), an increase in *NR3C1* mRNA levels significantly increased the odds of conversion, independently of cortisol, age, sex, cannabis use, and antipsychotic intake (OR = 2.03, p = 0.03), with an explained variance of 11.6% (pseudo-R²) (**Table 2**, **Figure 3**, **Supplementary Figure 3**).

**Table 2:** NR3C1 expression explains the risk of conversion independently of cortisol, age, sex, cannabis, and antipsychotic intake.

Variable	Odds Ratio	95% Confidence Interval	P-value
Intercept	0.52	[0.13‑2.19]	0.37
Sexe	0.41	[0.11‑1.49]	0.18
***NR3C1* mRNA**	**2.03**	**[1.08‑3.82]**	**0.03**
Cortisol at M0	0.97	[0.86‑1.08]	1.08
Age	0.85	[0.44‑1.63]	0.63
Antipsychotic use	0.95	[0.41‑2.19]	0.90
Cannabis in the last month	1.42	[0.85‑2.36]	0.18

P-values < 0.05 are in bold.

When applying the same combination of variables from model (I) to explain dimensional outcomes, no significant association was found between depression, or functional outcome, and *NR3C1* mRNA or cortisol, adjusted for age, sex, cannabis use, and antipsychotic intake.

### Genome-Wide Analysis Study of eQTL for *NR3C1* Expression, Stratified by Sex

After FDR correction, correction for stratification, and adjustment for age, cannabis, and antipsychotic intake, only one SNP, rs6849528, remained associated with *NR3C1* expression (p = 0.015, ES = 2.7) and explained 43% of its variance in the female group. The mean of *NR3C1* mRNA levels was robustly higher in subjects with at least one allele A (genotype AG or AA, 95% CI = [0.12‑0.15]) than in subjects with genotype GG (95%CI = [0.07 − 0.09]), with a significant non-parametric p-value = 2*10-4 (**Supplementary Figures 4** and **5**). No significant eQTL was found in males with the same correction and adjustments.

### eQTL-Based Mendelian Randomization

**Figure 4:**
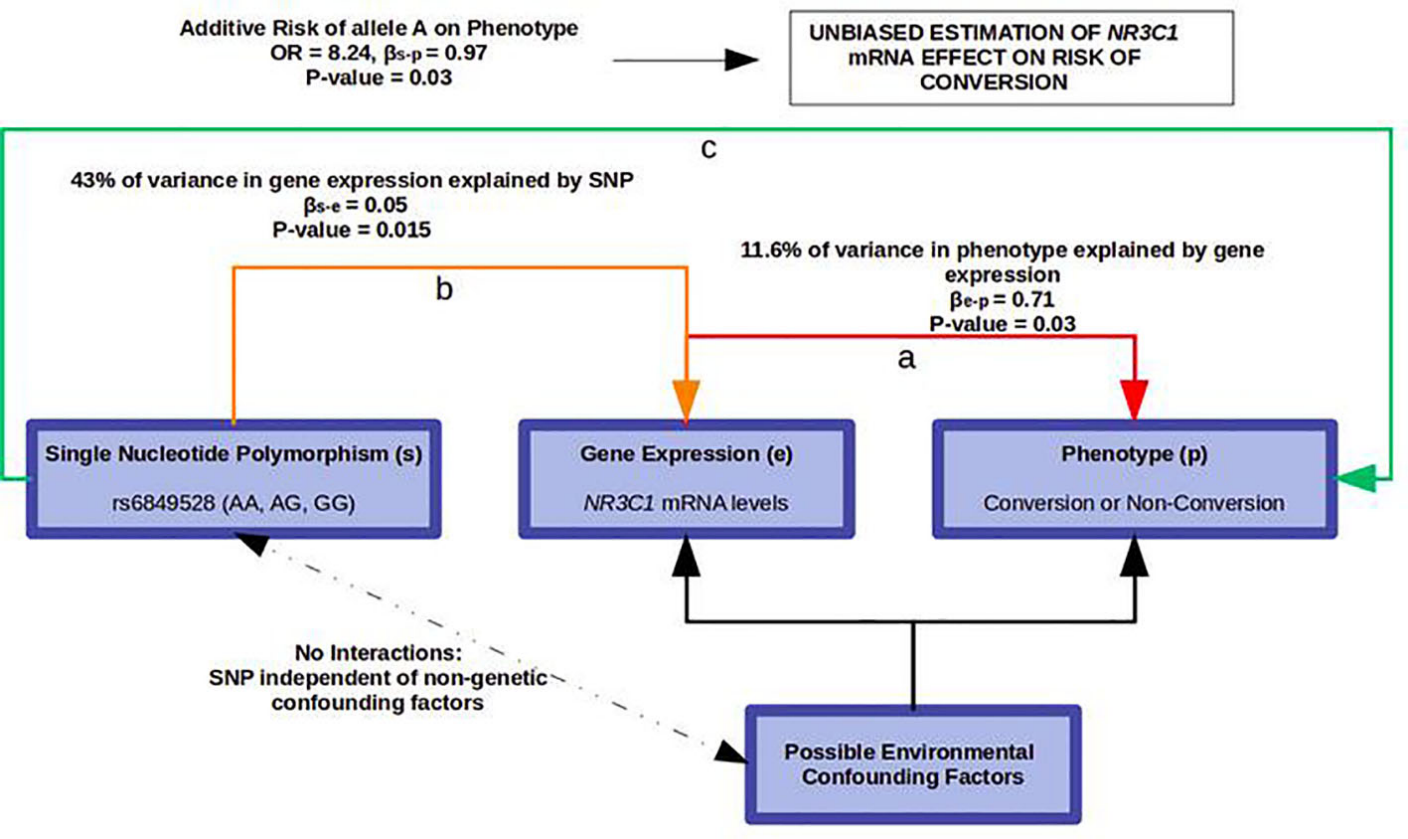
Mendelian Randomization using rs6849528 as an instrumental variable. a) NR3C1 mRNA levels are associated with psychosis. b) rs6849528 alleles lead to differential NR3C1 expression. c) Because the probability of distribution of allele A is independent of environmental factors, its association with psychosis is in favour of the involvement of NR3C1 mRNA in this risk, independently of non-genetic confounding factors.

rs6849528 could therefore be used as an instrumental variable for Mendelian randomization in the female group. Because the probability of distribution of its alleles is not related to environmental factors such as cannabis or tobacco use, randomizing the female cohort on rs6849528 would account for any unknown environmental confounder that could bias the association between *NR3C1* expression and conversion. Accounting for age, cannabis, and antipsychotic intake, the risk of conversion to psychosis was significantly associated with rs6849528, in an additive model, with an odds-ratio of 8.24 (p = 0.03) (**Figure 4**).

## Discussion

We applied multiple levels of analysis (hormonal, molecular and genetic) to test the association of conversion to psychosis with potential biomarkers from the biological stress pathway (*NR3C1* expression level and cortisol levels at different times of the day), while adjusting for age, sex, cannabis use, and antipsychotic intake, in a longitudinal cohort of 133 subjects at-risk for psychosis. Both stage IA and stage IB were included to be representative of daily clinical practice.

First, we showed that after adjusting for cortisol levels, an increase in *NR3C1* expression was significantly associated with a higher risk of conversion to psychosis. Cortisol itself was not associated with conversion, which is in line with our previous meta-analysis assessing the morning levels of cortisol (6). This negative result was extended to other times of the day (waking time, noon, afternoon). As cortisol levels were neither correlated with *NR3C1* expression, this may suggest cortisol’s effects in psychosis might be conditional to the underlying biological and genetic background.

Second, to strengthen the validity of *NR3C1* mRNA’s association with psychosis, we performed a Mendelian randomization analysis. This allowed to account for any possibly uncontrolled environmental confounding factor that could have biased the association. Because of reported *NR3C1* genotype-by-sex effects, we performed this GWAS eQTL analysis stratifying by sex. In females, we identified a SNP (rs6849528) strongly associated with *NR3C1* expression, and the odds of conversion to psychosis appeared significantly increased in the group with the minor allele A. Because the probability of distribution of alleles is independent of environmental factors, this SNP’s association with psychosis confirmed the involvement of *NR3C1* expression in the risk of psychosis, independently of any non-genetic confounder (**Figure 4**).

Whereas we initially postulated that the environment regulated gene expression, through the mediation of biological stress, we found that *NR3C1* expression, one of the genes most implicated in the stress pathway, was not dependent on cortisol levels, but rather on genetic variability, in females. Given the same level of cortisol, presuming the same level of stress, female individuals with higher *NR3C1* expression levels appeared thus more vulnerable than others to the risk of conversion.

The GWAS analysis did not find any significant eQTL associated with *NR3C1* expression in males, so Mendelian randomization could not be applied to bolster the association between *NR3C1* and psychosis in males. The absence of significant eQTL in males might either be due to a lack of power in a sample not large enough to detect the small effect-sizes of common polymorphisms, either to the aforementioned genotype-by-sex specificity of *NR3C1* regulation (16, 17).

In this Mendelian randomization framework, we used rs6849528 as an independent instrumental variable for randomization. This SNP, located in an intron of the acyl-CoA oxidase 3 gene (*ACOX3*) on chromosome 4, acted as a trans-eQTL regulator of *NR3C1*, located on chromosome 5. We wish to highlight the fact that we relied on its association with *NR3C1* expression, regardless of any actual pathophysiological mechanism, and with the unique purpose of randomizing the cohort in an environmentally unbiased way. It is indeed not possible to draw any conclusion regarding rs6849528’s real biological effect, especially as it may be associated with psychosis through a pleiotropic effect rather than causal association. ACOX3 participates in peroxisomal fatty acid beta-oxidation. Peroxisomal dysregulation has been described in relation with psychotic symptoms, in the context of inborn errors of metabolism (31), so a possible direct effect of ACOX3 gene on conversion to psychosis could not be ruled out. rs6849528 might affect *NR3C1* gene expression on the one hand, and phenotype, independently of *NR3C1* expression, on the other. In a purely causal model, the variation of gene expression (e) is fully determined by an eQTL SNP (s), which itself has no effect on phenotype (p) except through that gene’s expression, and βs-e x βe-p = βs-p (22). However, in our study, βs-e x βe-p << βs-p (0.04 < 0.97) suggesting pleiotropy. This was expected as psychosis is a complex phenotype resulting from the effect of many genetic and environmental factors. This also explained the limited variance of risk of conversion explained by *NR3C1* gene expression (11.6%) compared to the variance of gene expression determined by the eQTL (43%) (**Figure 4**).

Moreover, the causal biological effect may be driven by other SNPs in linkage disequilibrium with rs6849528, which could act as a proxy for its entire haplotype block in our eQTL analysis. None of the SNPs in rs6849528 haplotype block have been associated with schizophrenia in a GWAS. However, the haplotype block around rs6849528 comprises several SNPs acting as eQTLs of transfer RNA methyltransferase 44 (TRMT44), according to the GTEx portal (https://www.gtexportal.org/home/). One of these eQTL variants, rs6845969, impacts the expression of *TRMT44* in regions such as the hypothalamus or the pituitary gland, involved in the stress pathway. Other variants in this haplotype act as eQTLs for expression of *TRMT44* in the nucleus accumbens, relevant to motivation through reward and reinforcement (rs2386223; rs12503034, rs1880025). None of these SNPs were available for testing in our eQTL GWAS, but they could constitute pathophysiologically relevant factors; tRNAs have a major role in translation, decoding mRNA sequence into protein, and methylation of tRNA contributes to its stabilization (32). Conversely, hypomethylation leads to cleavage of tRNAs into tRNA-derived small RNA fragments, like microRNAs, which have been shown to activate stress pathways (33). This might explain how genetic variants could be associated with the expression levels of a distant mRNA (trans eQTL). Through their eQTL effect on TRMT44 activity, they might indirectly lead to an increase in specific miRNAs which in turn regulate gene expression. For instance, *NR3C1* has been reported to contain in its 3’ untranslated region a target of miR-124 (34).

Our study has several limitations. First, sampling times varied up to 1 h around the theoretical times for cortisol sampling. This could explain why cortisol measurements did not correlate with outcome. Second, a limitation inherent to Mendelian randomization analysis is that it is not possible to account for genetic confounders. In the same haplotype block, one SNP might only be associated with *NR3C1* expression level, while another might only correlate with phenotype. Their linkage disequilibrium would therefore act as a genetic confounding factor that cannot be disentangled by such analysis (35). Third, we only considered the variance of gene expression explained by the eQTL, while other non-genetic factors are also known to regulate *NR3C1* expression. For instance, CpG-specific methylation of *NR3C1* promoter regions has been robustly implicated in its expression and correlated with psychosocial stress (36). However, this restriction to genetic factors was necessary in order to ensure the unbiasedness of Mendelian randomization. Fourth, the eQTL analysis did not find any of the previously reported SNPs that are in the *NR3C1* gene (19). However, such studies used a candidate gene approach to analyze SNPs, while we looked for eQTL genome-wide. Any eQTL we found had to have therefore an important effect-size strengthening its interest for Mendelian randomization. Also, all the data came from peripheral measures: gene expression in blood and cortisol level in saliva. These data may imperfectly reflect the levels in brain tissue. However, blood-brain correlation has been reported to range from 0.25 to 0.64 and to be greater for genes highly expressed in both tissues (37). Finally, we cannot exclude that some non-converters at 1 year did convert later, but the conversion rate is maximal during the first year (3).

Further work is required to replicate our results, but our multimodal approach, using phenotypic, hormonal, transcriptomic and genetic data, illustrates how the implementation of statistical methods, e.g. with Mendelian randomization analyses, could help to detect biomarkers in psychiatry by adjusting for environmental factors. This also suggests that the impact of stress should be investigated in a comprehensive way, where cortisol levels would only be one of these factors.

## Data Availability Statement

The datasets presented in this article are not readily available because of an ethical issue. The consent form signed by the participants to the ICAAR study did not indicate that the genetic data would be shared or deposited in a repository.

## Ethics Statement

This study involved human participants and was reviewed and approved by Comité de protection des personnes, Ile-de-France III, Paris, France. Written informed consent to participate in this study was provided by the participants or their legal guardians.

## Author Contributions

AI, BC and M-OK designed the study. AI, OK, M-OK, GR, and BC obtained the funding and supervised the study. AI, OK, the ICAAR study group and BC collected the data. AI and BC analyzed the data. AI, OK, QH, TJ, GR, M-OK, and BC interpreted the data. AI, OK, and BC drafted the manuscript. All authors contributed to the article and approved the submitted version.

## Funding

This work has been supported by the French government’s “Investissements d’Avenir” programme, which is managed by the Agence Nationale de la Recherche (ANR), under the reference ANR-18-RHUS-0014 (“Project PsyCARE”). This work was also supported by the French Ministry grant PHRC AOM07-118 (for the ICAAR cohort), Institut National de la Santé et de la Recherche Médicale (INSERM), Université Paris Descartes (recurrent funding), the Canadian Institutes of Health research (GR), the Fondation Bettencourt Schueller (BC), and the Fondation pour la Recherche Médicale (AI). The Centre Hospitalier Sainte-Anne promoted the study. The sponsors had no role in the design and conduct of the study, in the collection, management, analysis or interpretation of the data, in the preparation, review or approval of the manuscript, or in the decision to submit the manuscript for publication. All the authors declare they have no competing interests related to this work.

## Conflict of Interest

The authors declare that the research was conducted in the absence of any commercial or financial relationships that could be construed as a potential conflict of interest.
