# Supplementary material for: Stress, Cortisol and NR3C1 in At-Risk Individuals for Psychosis: A Mendelian Randomization Study
Source: Front Psychiatry. 2020 Jul 10;11:680. doi: 10.3389/fpsyt.2020.00680 (PMC7367416; doi:10.3389/fpsyt.2020.00680)

**SUPPLEMENTARY DATA**

**Supplementary Table 1: Studies used as reference for the computation of chlorpromazine equivalents**

| Typical antipsychotic | Reference study |  |
| --- | --- | --- |
| Klorprotixene | (Kroken et al., 2009; Leucht et al., 2014) | ^1,2^ |
| Levomepromazine | (Kroken et al., 2009) | ^1^ |
| Trifluropromazine | (Davis, 1975) | ^3^ |
| Thioridazine | (Andreasen et al., 2010) | ^4^ |
| Dixyrazine | (Kroken et al., 2009) | ^1^ |
| Prochlorperazine | (Davis, 1975; Woods, 2005) | ^3,5^ |
| Perphenazine | (Andreasen et al., 2010) | ^4^ |
| Perphenazine decanoate | (Kroken et al., 2009) | ^1^ |
| Zuclopenthixol | (Kroken et al., 2009) | ^1^ |
| Zuclopenthixol decanoate | (Kroken et al., 2009) | ^1^ |
| Flupenthixol | (Kroken et al., 2009) | ^1^ |
| Flupenthixol decanoate | (Kroken et al., 2009) | ^1^ |
| Fluphenazine | (Andreasen et al., 2010) | ^4^ |
| Fluphenazine decanoate | (Andreasen et al., 2010) | ^4^ |
| Trifluoperazine | (Andreasen et al., 2010) | ^4^ |
| Acetophenazine | (Davis, 1975; Leucht et al., 2014) | ^2,3^ |
| Carphenazine | (Davis, 1975; Leucht et al., 2014) | ^2,3^ |
| Butaperazine | (Davis, 1975; Leucht et al., 2014) | ^2,3^ |
| Mesoridazine | (Davis, 1975; Leucht et al., 2014) | ^2,3^ |
| Piperacetazine | (Davis, 1975; Leucht et al., 2014) | ^2,3^ |
| Haloperidol | (Andreasen et al., 2010) | ^4^ |
| Haloperidol decanoate | (Andreasen et al., 2010) | ^4^ |
| Chlorprothixene | (Davis, 1975) | ^3^ |
| Thiothixene | (Andreasen et al., 2010) | ^4^ |
| Molindone | (Woods, 2005) | ^5^ |
| Prochlorperazine | (Leucht et al., 2014; Woods, 2005) | ^2,5^ |
| Atypical antipsychotics |  |  |
| Risperidone | (Andreasen et al., 2010) | ^4^ |
| Risperidone  action prolongée | (Kroken et al., 2009; Woods, 2005) | ^1,5^ |
| Olanzapine | (Andreasen et al., 2010) | ^4^ |
| Quetiapine | (Andreasen et al., 2010) | ^4^ |
| Ziprasidone | (Andreasen et al., 2010) | ^4^ |
| Aripiprazole | (Andreasen et al., 2010) | ^4^ |
| clozapine | (Andreasen et al., 2010) | ^4^ |
| Asenapine | (Leucht et al., 2014; Woods, 2005) | ^2,5^ |
| Iloperidone | (Leucht et al., 2014; Woods, 2005) | ^2,5^ |
| Lurasidone | (Leucht et al., 2014; Woods, 2005) | ^2,5^ |
| Paliperidone | (Leucht et al., 2014; Woods, 2005) | ^2,5^ |
| Sertindole | (Kroken et al., 2009; Leucht et al., 2014) | ^1,2^ |
| Amisulpride | (Bazire, 2007) | ^6^ |
| Sulpride | (Bazire, 2007) | ^6^ |

**Supplementary references:**

1. Kroken, R. A., Johnsen, E., Ruud, T., Wentzel-Larsen, T. & Jørgensen, H. A. Treatment of schizophrenia with antipsychotics in Norwegian emergency wards, a cross-sectional national study. BMC Psychiatry 9, 24 (2009).

2. Leucht, S. et al. Dose equivalents for second-generation antipsychotics: the minimum effective dose method. Schizophr. Bull. 40, 314–326 (2014).

3. Davis, J. M. Dose equivalence of the antipsychotic drugs. in Catecholamines and Schizophrenia 65–73 (Elsevier, 1975).

4. Andreasen, N. C., Pressler, M., Nopoulos, P., Miller, D. & Ho, B.-C. Antipsychotic dose equivalents and dose-years: a standardized method for comparing exposure to different drugs. Biol. Psychiatry 67, 255–262 (2010).

5. Woods, S. W. Calculation of CPZ Equivalents. at <www.scottwilliamwoods.com/files/Equivtext.doc> (2005).

6. Bazire, S. Psychotropic Drug Directory, Maudsley Guideline. (2007).

**Supplementary Figure 1 - *GAPDH* is a reference gene for *NR3C1* expression.**


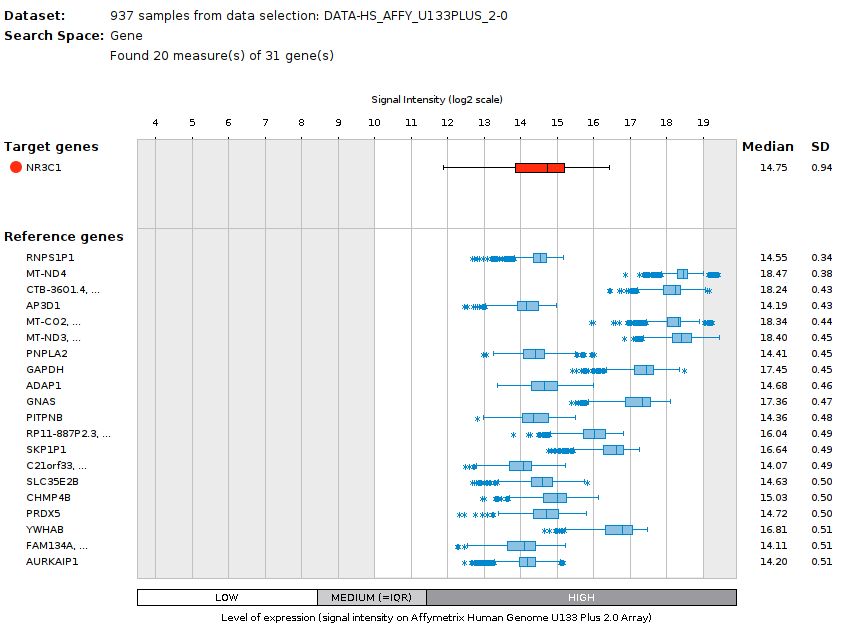


**Supplementary Figure 2 - Correlations between NR3C1 and cortisol measures in the male and female datasets.**
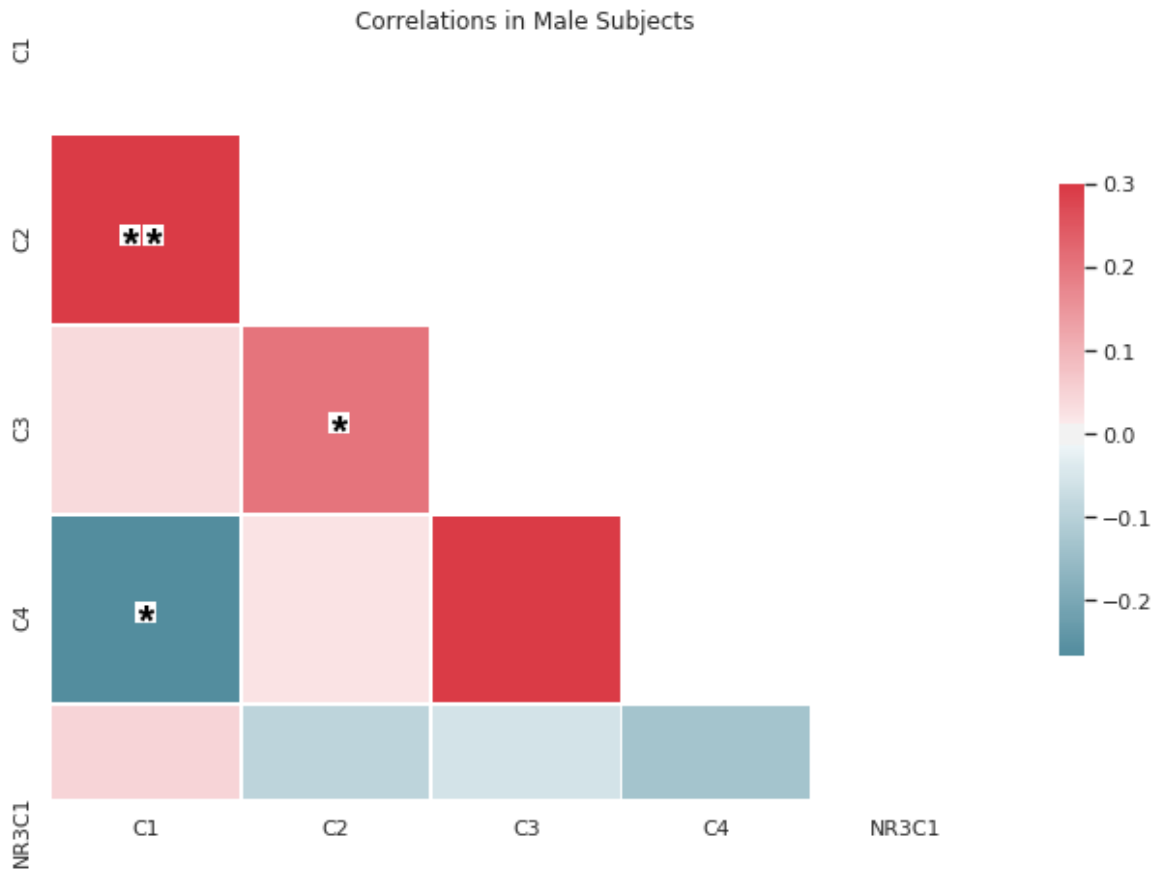

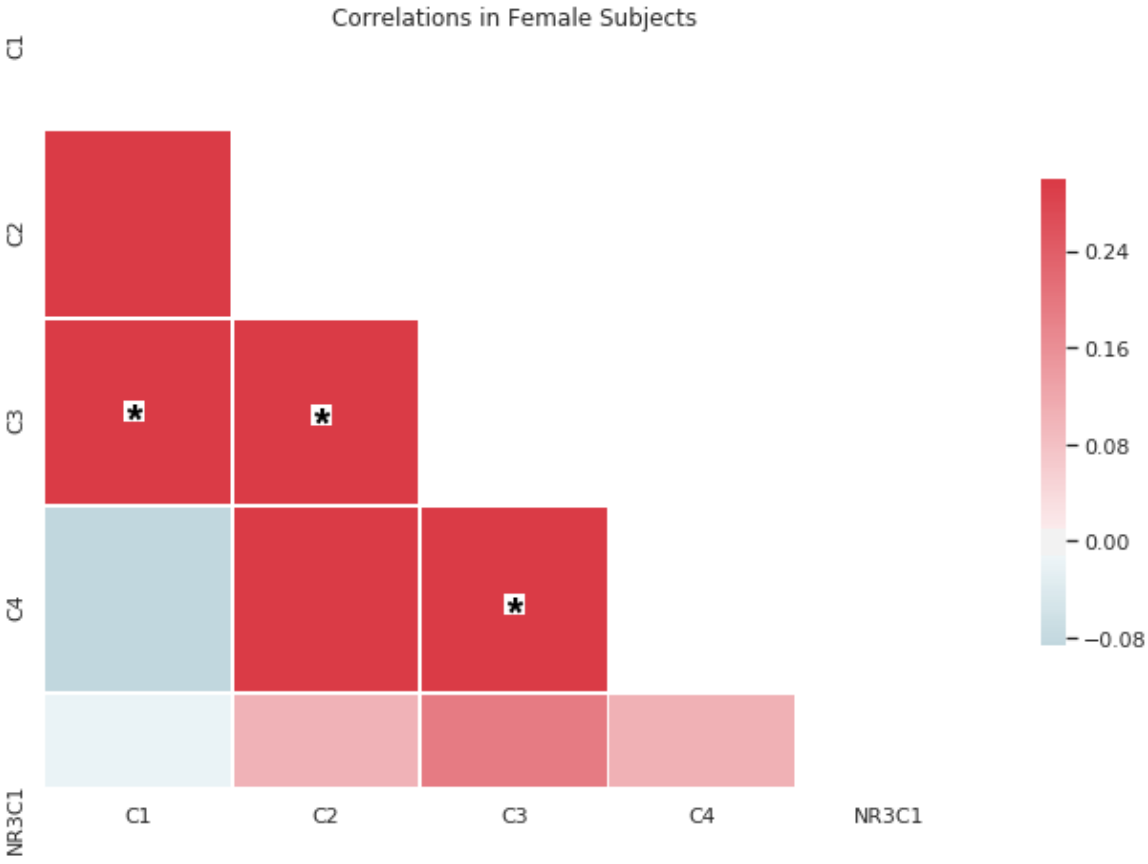


*** p-value < 0.05, ** p-value < 0.005, Spearman ρ correlation factor reported. Corrected p-value threshold (Bonferroni) on 15 tests in each group is 0.003. Cortisol measures weakly correlated with each other, and only a few were significant. Cortisol measures did not correlate with *NR3C1* gene expression.**

**Supplementary Figure 3**


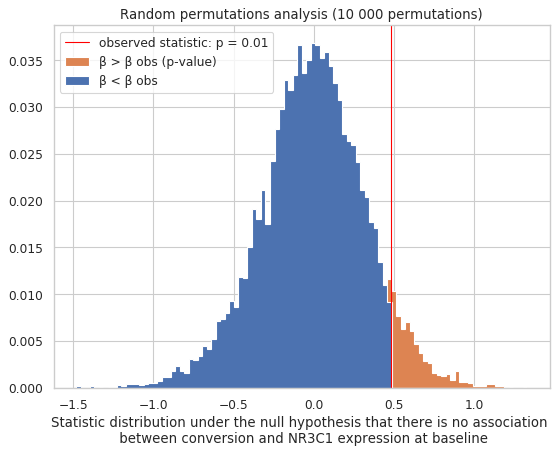


**Supplementary Figure 4: expression levels of NR3C1 depending on the rs6849528 genotype**


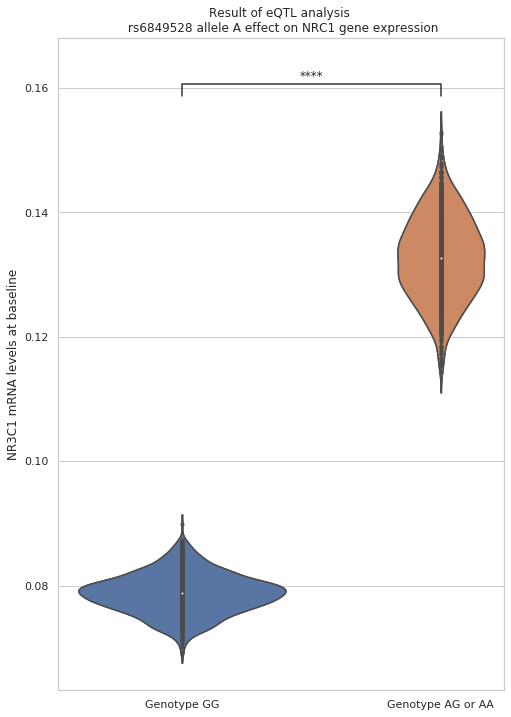


**Supplementary Figure 5**
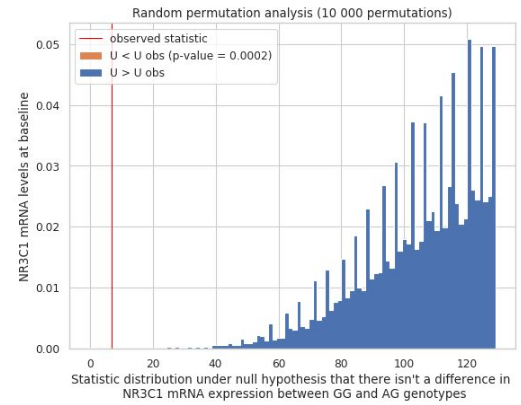

Supplement: Supplementary file 1 [file DataSheet_1.docx]
